# Re-visiting cognitive reserve: The importance of multiple brain measures

**DOI:** 10.1177/23982128261422282

**Published:** 2026-02-10

**Authors:** Richard N. Henson

**Affiliations:** 1 MRC Cognition and Brain Sciences Unit, University of Cambridge, Cambridge, UK; 2 Department of Psychiatry, University of Cambridge, UK

**Keywords:** Ageing, dementia, cognitive reserve

## Abstract

The term ‘cognitive reserve’ broadly refers to better-than-expected cognitive abilities in old age, presumed to reflect environmental/lifestyle factors earlier in life. This commentary addresses the question of what determines ‘better than expected’ cognition; specifically, whether cognitive reserve can be ‘explained away’ by considering multiple brain measurements. Using simulations, I show that, once one allows for multiple brain properties related to cognition, differential maintenance of those properties can reproduce the clinical picture associated with cognitive reserve. Using real data, I then show that white-matter microstructure and functional connectivity explain significant additional variance in fluid intelligence beyond grey-matter volume (at least cross-sectionally), supporting the importance of measuring multiple brain properties. Using multimodal, longitudinal data to identify changes in those brain properties that are especially important for changes in cognition will help decide which interventions are most likely to be effective at maintaining cognition in old age.

## Clinical definition of cognitive reserve

**Figure 1.**
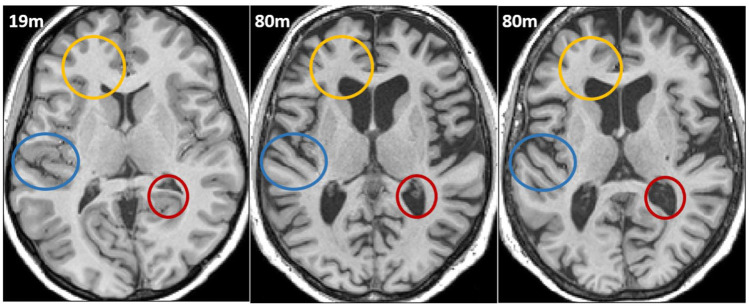
Horizontal sections through a T1-weighted magnetic resonance imaging (MRI) scan of 19-year-old male (left), and two healthy 80-year-old males (middle/right), which have been roughly aligned using a 12-parameter affine transformation. These individuals were selected from the Cam-CAN cohort (Shafto et al., 2014), such that one of the older males had a score on the Cattell test of fluid intelligence comparable to that of the young male, whereas the other older male scored much worse. Comparing the two older brains to the young one illustrates the dramatic ravages of time on brain structure: thinner cortical grey-matter (blue circles), less white-matter (yellow circles) and as a consequence, larger ventricles (red circles). Importantly, the scans illustrate the principle that two older brains can look very similar even if the ‘owner’ of one of them might be functioning well (e.g. living independently at home), while the owner of the other might struggle with typical age-related cognitive problems (e.g. requiring care). This puzzle has been called ‘cognitive reserve’, and underscores the fact that there must be more to brain structure and function than visible on this type of clinical scan.

The term ‘cognitive reserve’ has many uses in the literature, but for present purposes, it refers the observation that an individual’s cognitive abilities are better than would be expected from a standard clinical assessment of their brain. This is in the spirit of the original puzzle raised by (Katzman et al., 1988), who identified individuals whose cognitive abilities had been well above average, despite post mortem revealing neuropathology associated with Alzheimer’s Disease (see Valenzuela, 2019, for a historical perspective on the term). More commonly these days, the term is used by neurologists to describe people who show comparable atrophy on a clinical magnetic resonance imaging (MRI) scan, yet differ markedly in their cognitive function (Figure 1). This ‘puzzle’ is important, particularly if we can identify factors, such as lifestyle choices, that might boost this reserve, allowing individuals to maintain their cognitive abilities for longer, despite brain atrophy.

However, there have been theoretical critiques of the term cognitive reserve (Nilsson and Lövdén, 2018), along with debates about how to operationalise it (e.g. Cabeza et al., 2019), and despite attempts at a consensus definition (Stern et al., 2020), the term is still used inconsistently in the literature. One core issue is that cognitive reserve could simply reflect limitations of brain measurement, for example, insufficient information disclosed by a typical clinical, ‘structural’ MRI scan. This is the issue addressed here, where cognitive reserve is defined simply as that proportion of variance in cognition that cannot be explained by standard clinical brain properties, and the aim is to ‘explain away’ this puzzle by considering other brain properties. This resembles the ‘residual’ approach of Stern et al. (2020), and while this approach has been argued by Nilsson and Lövdén (2018) as unsatisfactory (because it means that cognitive reserve does not have theoretical definition independent of insufficient measurement), this matters less if cognitive reserve is simply a temporary short-hand for a concept that one seeks to explain away. (Note that this does not devalue the concept of cognitive reserve as a short-hand for the separate, though equally important, question of what lifestyle/environmental factors help maintain cognitive abilities in old age, but this is not the focus of the present commentary.)

Some have also distinguished cognitive reserve from ‘brain reserve’, where the latter could be, for example, simply the sheer number of neurons one possesses (such that one is more resilient to their loss), while the former could be, for example, psychological strategies used to maintain memory ability in older age. However, under the materialist assumption that our cognition depends solely on our brain, such psychological strategies would still have a functional brain correlate (when in use), in which case, once that correlate is measured, the distinction between brain reserve and cognitive reserve becomes specious. In other words, cognitive reserve must be explained by other brain properties. While not all such brain properties are currently measurable in vivo, there are many other types of brain scan, such as diffusion-weighted MRI (that can measure white-matter microstructure), functional MRI or electroencephalography/magnetoencephalography (EEG/MEG) (that can detect dynamic brain activity and connectivity), MRI spectroscopy (that can detect concentrations of some neurotransmitters), positron emission tomography (PET) (that can measure metabolic processes and abnormal proteins associated with neurodegenerative diseases) and MRI angiography (that can measure neurovascular health). Identifying at least some of these potential brain correlates will help illuminate the mechanisms of successful ageing and resilience to neurodegeneration, in turn guiding possible future interventions, for example, for lifestyle choices that might be associated with such mechanisms (e.g. vascular health).

## A developmental perspective on reserve versus maintenance

**Figure 2.**
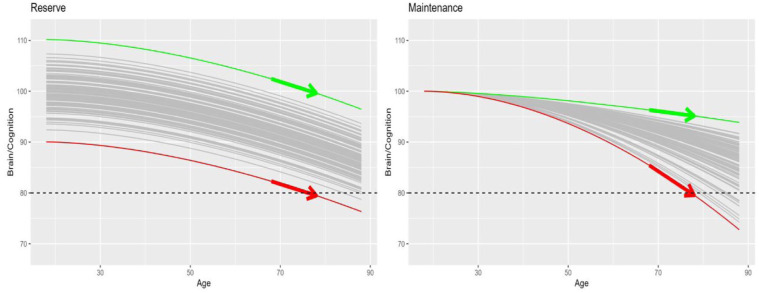
Reserve (left panel) versus maintenance (right panel) for a number of hypothetical individuals (grey lines). Y-axis could reflect cognitive ability (e.g. performance on an IQ test) or a measure of brain health (arbitrary units). Coloured lines represent lifespan trajectories of two individuals who differ most in reserve or maintenance, while the arrows indicate linear slopes across two measurement points. Black dashed line illustrates a hypothetical example cut-off for a cognitive test of dementia (again, units are arbitrary).

Some authors have distinguished between ‘reserve’ and ‘maintenance’ in developmental terms (Nyberg et al., 2012; Walhovd et al., 2023), where reserve refers to a time-invariant difference between adults, whereas maintenance refers to a difference that emerges with age (Figure 2). These are generic labels that could apply to brain, cognition or any other property that changes across the lifespan. Tucker-Drob (2019) made a related distinction between ‘preserved differentiation’ and ‘differential preservation’ of cognition, and gave examples of the effect of education, which appears to function like reserve throughout adulthood (preserved differentiation), and the APOE genotype, which appears to affect maintenance in late life (differential preservation). In terms of the brain, reserve might be determined by early-life factors that affect, for example, cortical surface area, while maintenance might relate to later life-style factors (e.g. physical activity in middle age) that moderate the rate of decline in, for example, cortical thickness. While not everyone agrees with this restricted use of the term ‘reserve’ (as a stable difference between individuals across time), it is useful to illustrate the terminological confusion that results when contrasted with the above clinical definition of cognitive reserve, as explained later.

Note that distinguishing reserve and maintenance requires measuring a cognitive or brain property across more than one time-point (i.e. requires longitudinal data): reserve predicts no difference in slope between individuals, whereas maintenance predicts a steeper slope in those less able to maintain their brain/cognitive health (indicated by arrows in Figure 2). This has important implications for dementia. If a dementia diagnosis is (at least partially) dependent on someone’s performance on a standard cognitive test (e.g. the Mini-Mental State Examination), with a hypothetical cut-off shown by the dashed line in Figure 2, then the ‘red’ person is more likely to receive a dementia diagnosis in later life than the ‘green’ person. However, if the differences between these two people reflect reserve, then the red person may have always done poorly on cognitive tests, and their rate of decline is actually no faster than that of the green person. This suggests that the red person does not have a progressive degenerative disease like Alzheimer’s (that emerges later in life), since that would predict a pattern more like the maintenance case, that is, they should show a faster-than-average decline instead. Of course, faster decline is not sufficient to infer a neurodegenerative disease, since normal genetic variation (like in APOE) could also lead to different rates of decline in late life (e.g. if the trajectories in Figure 2 were from a projectile, then genetics could cause variation in its initial horizontal velocity); additional biomarkers are normally needed for such a diagnosis. The important point here is that a single measurement at one time-point cannot distinguish the two possibilities in Figure 2.

## Confusion between developmental reserve and cognitive reserve

**Figure 3.**
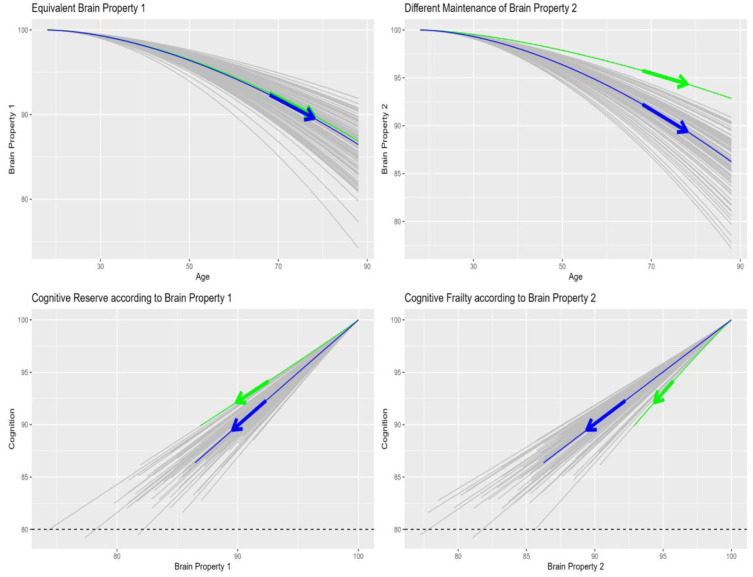
Illustration of clinical concept of cognitive reserve. The top two panels show two hypothetical brain properties (e.g. grey-matter volume and white-matter microstructure), the first of which shows equivalent decline with age for two individuals, while the second shows better maintenance for the green person than the more typical blue person. Cognitive reserve is apparent in the bottom left plot, where the green person has better cognitive ability than would be expected from their first brain property (e.g. grey-matter volume from a standard clinical MRI scan like in Figure 1). The corollary of this is that they would have worse cognitive ability than would be expected from their second brain property (bottom right plot). Note that the blue person follows the main diagonal in both bottom panels because they possess equivalent and typical age-trajectories for both brain properties (in top panels). Arrows show slopes between the same two time-points (ages) shown in Figure 2. R markdown code for these simulations is provided in https://github.com/RikHenson/CogRes.

Nonetheless, the clinical concept of Cogntive Reserve refers to a relationship between cognitive measures and brain measures, not between cognition/brain and age. Importantly, cognitive performance may depend on more than one brain property. This is illustrated in Figure 3, where cognition was simulated as the simple average of two brain properties. The first brain property shows equivalent age trajectories for the green and blue person (top left panel), while the second shows better maintenance for the green person. For example, the first property might be grey-matter volume, which is a common index of brain health from a standard clinical scan (i.e., can be derived from MRI images like those in Figure 1). The second property might instead relate to micro-structural properties of white-matter (as revealed by diffusion-weighted MRI, but not visible on the typical clinical scan in Figure 1), which the green person is better able to maintain for some reason (due to higher levels of physical activity for example).

Cognitive reserve is then illustrated in the bottom left panel, which plots cognitive performance against the first brain property (e.g. grey-matter volume). As individuals get older, their cognitive and brain health both decline (shift towards bottom left of plot). However, the green person shows higher cognitive ability, and slower decline, than would be expected from their first brain property, that is, evidence of cognitive reserve. This is because their cognitive ability also includes a contribution from the second brain property, which is higher than typical (e.g. higher than the more typical blue person). In other words, another brain property of the green person (such as white-matter microstructure) “compensates” for their typical loss of grey-matter volume, explaining why they are functioning better than would be expected from a standard clinical scan.

In practice, the multiple properties of each person’s brain are likely to be correlated. For example, Wallerian degeneration might cause parallel loss in both grey-matter and white-matter (and likewise for functional connectivity, neurotransmitter loss, neurovascular health, etc.). Indeed, in the simulations in Figure 3, the rates of change of the two brain properties were correlated at 0.5. However to the extent that these properties are at least partially dissociable, it is still possible to find people whose brain health according to one property (e.g. white-matter microstructure) is unusually high relative to another (e.g. grey-matter volume), and these are the people who will be classed as having cognitive reserve according to a clinical MRI.

Note that this simple model conforms to the general concept of brain maintenance, where higher cognitive abilities will be associated with ‘younger-looking’ brains (Nyberg et al., 2012). Importantly however, it raises the possibility that one might maintain one brain property, but not another, which renders predictions for cognition harder to make, particularly if some properties are not measured. Moreover, in relation to terminology, the above simulation is an example where *brain maintenance* leads to *cognitive reserve*. The failure to make this distinction between (1) effects of age on cognition and (2) effects of brain on cognition has contributed to the terminological confusion in this field.

## Evidence for the importance of multiple brain properties

**Figure 4.**
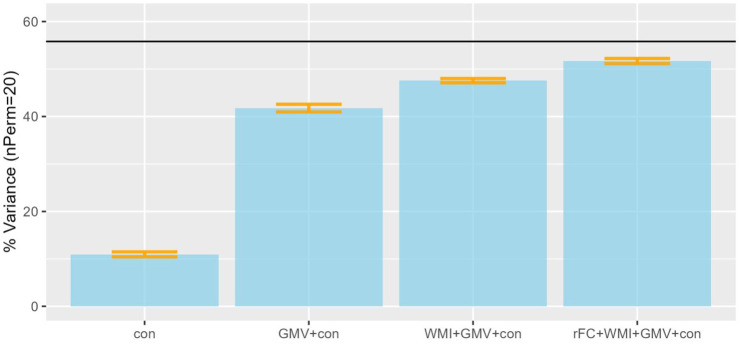
The proportion of variance in fluid intelligence explained by various brain metrics of N = 586 adults aged from 18 to 88 in the Cam-CAN Stage 2 data (www.cam-can.org). The horizontal line is an estimate of the ‘noise-ceiling’, that is, maximum explainable variance based on reliability of the Cattell test of fluid intelligence (estimated from an independent cohort). Variance explained was estimated using 10-fold, nested cross-validation of a linear model with L1- and L2-regularisation, with error bars from 20 permutations. The left bar reflects potential confounds (con), such as sex and polygenic score for intelligence. The next three bars reflect variance explained after stacking predictions from additional: (1) grey-matter volume (GMV) from 98 ROIs in the Harvard-Oxford atlas after segmenting a T1-weighted MRI; (2) white-matter integrity (WMI) from 48 ROIs in the Julich atlas using mean signal kurtosis from a Diffusion-weighted MRI; (3) resting-state connectivity (rFC) from 153 connections within and between 17 Yeo networks from BOLD-weighted MRI. Of course, it is possible that multiple brain properties interact in determining cognition, which could be modelled by adding multiplicative relationships. Data and code are available from https://github.com/RikHenson/CogRes

While these simulations were made to clarify the concept of cognitive reserve, is there actually evidence that multiple brain properties make independent contributions to cognitive abilities? Figure 4 provides some evidence, using data from the Cam-CAN cohort (Shafto et al., 2014). The y-axis shows the percentage of variance explained in the Cattell test of fluid intelligence as a function of different combinations of brain measures. Fluid intelligence captures the ability to reason quickly in novel situations, which underlies performance on nearly all cognitive tests (Spearman, 1927) is strongly predictive of everyday function (Diehl et al., 2005), and importantly is known to decline rapidly with age.

The first bar in Figure 4 shows variance in fluid intelligence explained by potential ‘confounds’ (around 11%) like sex and polygenic score for intelligence. This is a relatively small proportion of the potentially explainable variance (around 56%; horizontal black line), that is, taking into account measurement error in the Cattell test. The second bar shows the proportion explained by grey-matter volume (around 42%), analogous to what might be achieved from a clinical MRI scan (though using machine learning methods across approximately 100 distinct brain regions, rather than just visual assessment). The third bar shows a small but significant increase in variance explained (to around 48%) when adding estimates of the integrity of major white-matter tracks as estimated from diffusion-weighted MRI. The final bar shows yet further improvement (to around 52%) when adding functional connectivity estimates from resting-state BOLD-weighted fMRI. This is preliminary evidence that different brain properties offer some degree of complementary information about cognitive ability.

Much of the variance in fluid intelligence and in brain measures in Figure 4 reflects the wide range of ages in CamCAN (18-88), and it is possible that the covariance between them is an artefact of independent effects of age on all the variables. Despite the likelihood that the brain properties do in fact mediate a reasonable proportion of the effects of age on cognition, the possibility of independent age effects is difficult to refute from cross-sectional data alone. This is where longitudinal data are again important, to confirm that age-related change in each of the brain measures covaries with change in cognitive abilities. Few cohorts exist with multi-modal, longitudinal brain data, but several groups are now collecting (and sharing) such data.

## Conclusion

In summary, one clinical definition of cognitive reserve refers to a dissociation between cognitive ability (or everyday functioning) and brain health according to a standard clinical brain scan. This is related to, but different from, developmental concepts of reserve and maintenance that relate cognitive or brain health to age. Recent advances in brain imaging techniques offer complimentary windows on brain health, to the extent that we may be able to explain away more and more of the puzzle of cognitive reserve. In particular, future research needs longitudinal and multiple measures of brain health, to see which brain properties make independent contributions to changes in cognition. Identifying the contributions to cognitive change from brain properties other than standard grey-matter volumetrics, such as white-matter integrity, functional activity, functional connectivity, neurotransmitter concentrations and vascular function, offers the potential to understand the mechanisms through which possible interventions, like changes in lifestyle, might allow people to maintain cognitive abilities for longer in the presence of the inevitable brain changes associated with ageing and neurodegeneration.
